# Accurate and Rapid Estimation of Phosphene Thresholds (REPT)

**DOI:** 10.1371/journal.pone.0022342

**Published:** 2011-07-22

**Authors:** Arman Abrahamyan, Colin W. G. Clifford, Manuela Ruzzoli, Dan Phillips, Ehsan Arabzadeh, Justin A. Harris

**Affiliations:** 1 School of Psychology, The University of Sydney, Sydney, New South Wales, Australia; 2 Cognitive Neuroscience Section, IRCCS San Giovanni di Dio Fatebenefratelli, Brescia, Italy; 3 The Magstim Company Ltd, Whitland, Wales, United Kingdom; 4 School of Psychology, The University of New South Wales, Sydney, New South Wales, Australia; University of Bologna, Italy

## Abstract

To calibrate the intensity of transcranial magnetic stimulation (TMS) at the occipital pole, the phosphene threshold is used as a measure of cortical excitability. The phosphene threshold (PT) refers to the intensity of magnetic stimulation that induces illusory flashes of light (phosphenes) on a proportion of trials. The existing PT estimation procedures lack the accuracy and mathematical rigour of modern threshold estimation methods. We present an improved and automatic procedure for estimating the PT which is based on the well-established Ψ Bayesian adaptive staircase approach. To validate the new procedure, we compared it with another commonly used procedure for estimating the PT. We found that our procedure is more accurate, reliable, and rapid when compared with an existing PT measurement procedure. The new procedure is implemented in Matlab and works automatically with the Magstim Rapid^2^ stimulator using a convenient graphical user interface. The Matlab program is freely available for download.

## Introduction

Transcranial magnetic stimulation (TMS) is a non-invasive brain stimulation method using a coil placed next to the intact scalp. The magnetic field generated by the coil passes unimpaired through the scalp and the skull and stimulates the underlying cortex [1]–[4]. The dose of magnetic field required to effect this stimulation varies between individuals and thus requires individual calibration [5]. While there are established procedures for calibrating TMS intensities for the motor areas of the brain (motor threshold), there are no well-defined procedures for calibrating magnetic field intensities when applied to the occipital cortex (phosphene threshold, PT). To fill this gap, we propose an automatic procedure for estimating the PT which is rapid, accurate, and reliable when compared with another commonly used procedure for estimating the PT. We named our procedure REPT, which stands for “rapid estimation of phosphene thresholds”. The procedure is implemented in Matlab and available for download from http://www.psych.usyd.edu.au/tmslab.

The effects of TMS depend on a variety of factors, including stimulated brain area, coil type [6], pulse waveform [7], current direction [8], as well as stimulation parameters involving frequency, number of pulses, and TMS intensity [9]. TMS intensity, which is commonly defined as a percentage of stimulator's maximum output, is particularly important. Even if all other stimulation parameters are kept identical between participants, individual differences in cortical excitability, cortical structure and skull shape (which affects the distance from the scalp to the cortex [10], [11]) can significantly influence the TMS dose that is necessary to achieve comparable levels of cortical excitability between participants. TMS intensities therefore require individual calibration to ensure comparable neurophysiological effects between participants.

There are three main approaches to choosing TMS intensities. One simple approach is to select a fixed intensity for all participants and avoid individual calibration. Several studies have successfully used this approach to demonstrate consistent behavioural effects [12]–[15]. Choosing single intensity for all participants reduces the time necessary to calibrate TMS intensities individually. However, this approach is unsuitable for studies in which stimulation above or below calibrated thresholds can produce different neural [16] or behavioural outcomes [17]–[19] and thus where individual calibration is required.

A commonly used approach to calibrating TMS intensities involves measurement of the motor threshold (MT) [9], [20]. MT is estimated by delivering pulses of varying intensities over the primary motor cortex and measuring evoked muscle contractions in the somatotopically related part of the body. The (resting) MT is defined as a minimum TMS intensity that elicits motor evoked potentials (MEPs) of predefined amplitude (typically 50 µV) on a certain proportion of trials [21]. Pulse intensities for an experiment can then be set at a percentage of MT to ensure that stimulation produces equivalent effects between participants.

The MT has been shown to be unsuitable as a measure of cortical excitability for more posterior regions of the brain such as the occipital cortex ([22]–[24], but see [25]).

For such purposes, a better alternative has been identified to be the visual PT. The phosphene is an illusory visual percept that can be elicited by applying TMS to human visual cortex [26], [27]. The PT is the level of stimulation that induces phosphenes on a certain proportion of trials.

Some of the procedures for measuring the MT have been adopted for measuring the PT, including the method of constant stimuli (MOCS) [8], a truncated version of the method of limits (the Rossini-Rothwell procedure [21], [28], used in [22], [29]), and the modified binary search algorithm (MOBS [30], [31], used in [32]–[34]). These procedures, however, have limitations. MOCS, while accurate, is often impractical for establishing PTs on a regular basis because it requires many trials, greatly increasing the set-up time before the start of the experiment. The Rossini-Rothwell method, while fast, has a high degree of variability [35] and this can be inadequate for studies that require more reliable measures of the PT [8], [17]. Finally, the MOBS procedure, while systematic and fast, lacks a theoretical foundation as it uses heuristically determined rules when estimating thresholds [36]. To address these issues, we have implemented the REPT procedure, that uses a well-established Bayesian adaptive staircase protocol, Ψ, for estimating psychophysical thresholds [37].

The Ψ Bayesian adaptive staircase employs a sophisticated approach in choosing stimulation intensities to efficiently converge on a threshold. The computed threshold has been shown to be accurate and stable when compared with the “true measure” of the threshold [37], [38]. After each response, the Ψ procedure updates a posterior distribution across a set of psychometric curves which cover a broad sampled space of stimulus thresholds and slopes. The upcoming stimulus is computed across the posterior probability space to select the stimulation intensity which minimises the entropy (or uncertainty) as to which one is the actual psychometric function corresponding to the participant's performance. The threshold is always estimated in 30 trials and takes a little longer than a minute to run, thus providing both the participant and experimenter with the certainty about the duration of the procedure.

One of the bottlenecks of the existing procedures for estimating PTs is that they are usually performed manually: on each trial, the experimenter uses a control panel on the TMS machine to set the stimulation level. The manual approach introduces an opportunity for human error [39], is slow, and requires verbal responses which can cause head movement and thus affect the coil position and interfere with the accuracy of stimulation. Moreover, while existing procedures are computationally simpler and can afford quick calculation and manual adjustment of stimulation intensity, the Ψ procedure requires complex computations on each trial and therefore can benefit from interfacing with the stimulator to programmatically change the stimulation intensity. To address all these limitations, we have developed a Matlab toolbox, called Rapid2, to control the Magstim Rapid^2^ stimulator via a serial interface to programmatically change the stimulation intensity. REPT employs the Rapid2 toolbox to automatically change the pulse intensity and deliver a pulse from a computer. Both REPT procedure and Rapid2 toolbox are freely available for download from http://www.psych.usyd.edu.au/tmslab (see also [40], [41] for other programmatic approaches to using Magstim stimulators).

We validated REPT by contrasting it with the MOBS procedure [32]–[34]. The validation of accuracy was performed by comparing thresholds from REPT and MOBS with thresholds obtained using the method of constant stimuli (MOCS), which was used as a measure of the true threshold. The reliability of REPT and MOBS was evaluated by computing the variability in thresholds collected across multiple runs. We also compared the speed of REPT and MOBS by comparing the duration of each procedure.

## Methods

### Participants and Setup

We recruited and obtained informed written consent from 10 healthy participants (age range 24–43; five females) who were naive to the goals of the study. They were screened according to the TMS safety guidelines [42], [43]. All selected participants reported clear perception of visual phosphenes. The study was approved by the Human Research Ethics Committee at the University of Sydney.

Participants were stimulated with the Magstim Rapid^2^ stimulator (Withland, UK, www.magstim.com) using a 70-mm figure-of-eight coil. We used a real-time neuronavigation system (Softaxic, EMS Medical, Italy and Polaris Vicra, NDI Medical, USA) which helped to ensure consistent positioning of the coil at the target brain area throughout all thresholding procedures.

### Procedure

Participants were seated in a dimly lit room and were allowed at least 5 min to adapt to this level of illumination. With eyes closed, participants were asked to fixate the remembered position of a cross on a monitor directly in front of them. They were also asked to be vigilant to the presence of phosphenes, to ignore the intensity of the auditory click accompanying the pulse when judging phosphenes, and to report the presence or absence of phosphenes. When unsure of seeing a phosphene, participants were instructed to respond “no”. Five participant (AH, HM, IM, NX and WYC) had no previous experience of visual phosphenes and were provided with a description and experience of phosphenes prior to the experiment.

The coil was positioned with the handle pointing to the left side of the participant, parallel to the ground. Initially, the coil was placed with the centre over an area 3 cm above the inion and 2 cm lateral. Single pulses were delivered with intensities reaching 70% while the coil was moved in steps of 0.5 to 1 cm assisted by neuronavigation. The position of the coil that evoked bright and reliable phosphenes was marked as a “hotspot”. The coil was fixed at the hotspot with a clamp and articulated arm and the coil position was recorded within the neuronavigation software which helped to monitor the coil position with 2 mm precision throughout the experiment. For nine out of ten participants, phosphenes were elicited by stimulating the left occipital lobe, while for one of the participants (AH) the stimulation was delivered over the midline.

REPT and MOBS were run in consecutive blocks and we collected 4 measures of threshold for each procedure. The order of REPT and MOBS blocks was counterbalanced across participants. We also recorded the duration of each staircase.

### REPT

The pulse was delivered automatically from the computer. Participants were instructed to respond to the presence or absence of phosphenes using the Right or Left “Shift” key on a computer keyboard.

### MOBS

Pulse intensities for MOBS were adjusted and delivered manually from the stimulator. This was done to emulate threshold estimation as typically carried out in other studies, and thus to compare with an automatic procedure of estimating the PT in terms of accuracy, reliability and speed. When estimating the PT using MOBS, participants were asked to respond to the presence or absence of phosphenes by saying “yes” or “no” aloud. Each response was entered into an Excel sheet which calculated the next pulse intensity and the TMS intensity was set by turning a dial on the stimulator. The range of intensities for MOBS was set from 1% to 100% of stimulator output. The experimenter manually adjusted the pulse intensity, and after giving a warning to the participant, delivered the pulse. The MOBS threshold was estimated after six reversals (i.e., six changes from seen to unseen or vice versa) [30].

### MOCS

The pulse intensity and delivery was controlled automatically from the computer, similar to REPT; participants reported detecting phosphenes using either the Right or Left “Shift” key on a computer keyboard. The MOCS procedure was always run last because threshold estimates from REPT and MOBS helped in selecting the range of stimulation intensities to test (otherwise, we would have had to test each participant with many more levels of TMS intensity to be sure to include the relevant range around the PT). We selected seven pulse intensities for MOCS that were approximately centred around the assumed location of the participant's threshold (the only exception was participant LS, for whom we used six pulse intensities). The selected pulse intensities covered the range of phosphene detection accuracies ranging from 0% to 100%, and each pulse intensity was randomly presented 20–30 times within a session.

### Data Analysis

In REPT, the estimated PT corresponds to the position parameter of a Weibull function [44] fitted to the proportion of phosphene responses. REPT uses the Weibull function because it is well suited to model a wide variety of psychometric data [45], [46], particularly for contrast detection [47]. When using the Weibull function to model psychometric functions, researchers typically define the threshold (or the point of subjective equality) at a value corresponding to 63% correct, rather than 50%, due to the asymmetric nature of the Weibull function. In REPT, we also apply a correction to account for lapses on 4% of trials [48]. After the correction, the final threshold provided by the REPT procedure corresponds to 60% accuracy. Note that it is possible to calculate 50% post hoc threshold in REPT using the threshold and slope values of the psychometric function estimated using the Ψ staircase. We have added this calculation to the REPT interface. However, the 50% threshold can be less accurate than the 60% threshold because accurate estimation of the slope requires 300 trials, whereas the 60% threshold can be accurately and reliably calculated after just 30 trials [37]. Therefore, we advise using 60% threshold provided by REPT.

To determine thresholds from MOCS, the data were fitted with a cumulative Weibull psychometric function via a maximum likelihood criterion using the Palamedes toolbox [49]. Thresholds were computed for 60% and 50% response accuracy to match threshold estimates of REPT and MOBS, respectively. Note that the reported thresholds refer to the percentage of Magstim Rapid^2^ stimulator's maximum output (1.2T in the case of 70 mm figure-of-eight coil), unless otherwise indicated.

## Results

**Figure 1:**
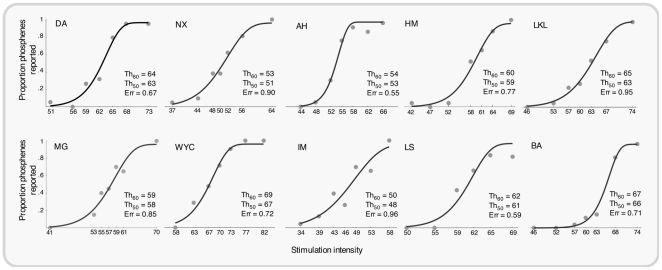
Proportion of phosphene detection for different levels of TMS intensity using the method of constant stimuli for 10 participants. The data were fitted with a cumulative Weibull psychometric function to obtain 60% and 50% phosphene thresholds. Stimulation intensity refers to the percentage of stimulator's maximum output. Error values indicate threshold estimation errors using parametric bootstrapping procedures.

**Figure 2:**
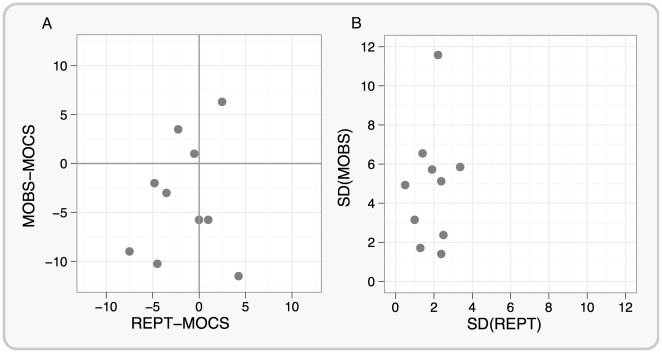
Comparisons of REPT and MOBS performance in terms of accuracy (Panel A) and variability (Panel B) for 10 participants. Panel A shows the difference between REPT and MOCS on the horizontal axis and the difference between MOBS and MOCS on the vertical axis, for each participant. It is clear that the REPT-MOCS values are closer to zero than are the MOBS-MOCS values, as confirmed by the fact that the absolute difference between REPT and MOCS was significantly smaller than the absolute difference between MOBS and MOCS. This suggests that REPT thresholds were more accurate than MOBS thresholds. Panel B displays the relationship between the standard deviation of REPT and MOBS thresholds for each participant. The variability of phosphene thresholds measured with REPT was significantly lower than the variability of phosphene thresholds measured with MOBS which suggess that REPT thresholds were more consistent.

The cumulative Weibull psychometric functions fitted to the MOCS data for each participant are shown in Figure 1. To assess the accuracy of REPT and MOBS procedures we compared them with MOCS which provides highly accurate threshold estimates (true thresholds). The comparison scores were the absolute value differences in the mean of four staircases for REPT or MOBS from MOCS. We found that the absolute differences between REPT and MOCS (*M* = 3.08, *SD* = 2.29) were smaller compared with the absolute differences between MOBS and MOCS (*M* = 5.80, *SD* = 3.55), *F*(1,9) = 7.14, *p* = .025, indicating that REPT provided more accurate threshold estimates than MOBS (the scatterplot of individual results is provided in Figure 2A). We also compared the reliability of REPT and MOBS by computing the standard deviation from the four thresholds that were collected for each procedure. Overall, REPT had lower variability (*M*(σ) = 1.90) than MOBS (*M*(σ) = 4.84), *F*(1,9) = 9.65, *p* = .01, which suggests that threshold estimates obtained using REPT were more reliable than MOBS. The scatterplot of individual standard deviations between each method is shown in Figure 2B.

**Figure 3:**
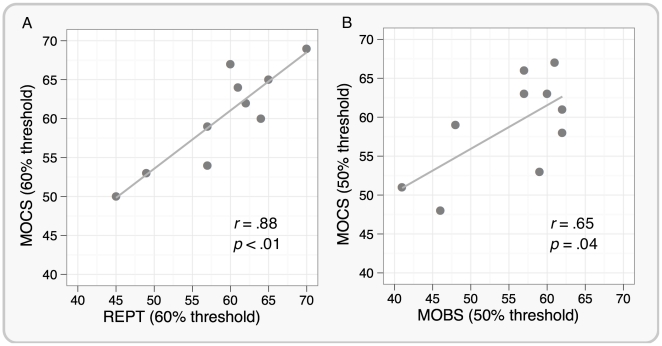
Scatterplot between REPT and MOCS (Panel A), and MOBS and MOCS (Panel B) phosphene thresholds for 10 participants. Both REPT and MOBS thresholds well correlated with the phosphene thresholds estimated using MOCS. However, the relationship between REPT and MOCS appears stronger than between MOBS and MOCS.

There was no overall difference between REPT and MOCS thresholds (*t*(9) = 1.33, *p* = .22), or between MOBS and MOCS thresholds (*t*(9) = 1.9, *p* = .08), showing that neither REPT nor MOBS produced any systematic over- or underestimate of the PT relative to that provided by MOCS. There was a significant correlation between REPT and MOCS (*r* = .88, *p*<.01) as well as between MOBS and MOCS (*r* = .65, *p* = .04), as confirmed by scatterplots shown in Figures 3A and 3B, respectively. However, it appears that the relationship between REPT and MOCS is stronger compared with the relationship between MOBS and MOCS.

**Figure 4:**
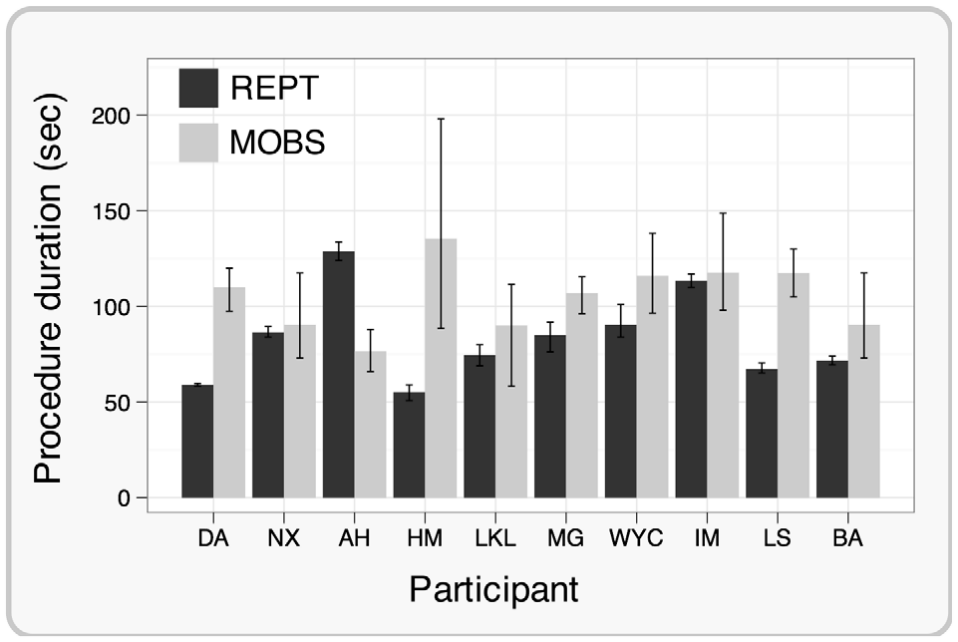
The duration of REPT and MOBS procedures. Panel B shows the average duration of the REPT and MOBS procedures for each participant. The duration of the REPT was significantly shorter compared with the duration of the MOBS. The duration of REPT was also more predictable, compared with MOBS, because it always finished in 30 trials, while the number of trials in MOBS varied considerably. Error bars represent 95% bootstrapped confidence intervals.

To assess the efficiency of REPT and MOBS, we compared the time taken to estimate a threshold with each procedure. On average, REPT was 1.3 times faster than MOBS and this speed advantage was significant (*F*(1,78) = 12.35, p<.01). REPT was also more predictable because it always finished in 30 trials and lasted on average 83 sec with very little time variability between each run (*M*(σ) = 3.02 sec). MOBS, on the other hand, lasted on average 105 sec and there was considerable within-subject variability of the procedure duration (*M*(σ) = 16.83 sec). Individual mean durations of REPT and MOBS procedures are shown in Figure 4.

**Figure 5:**
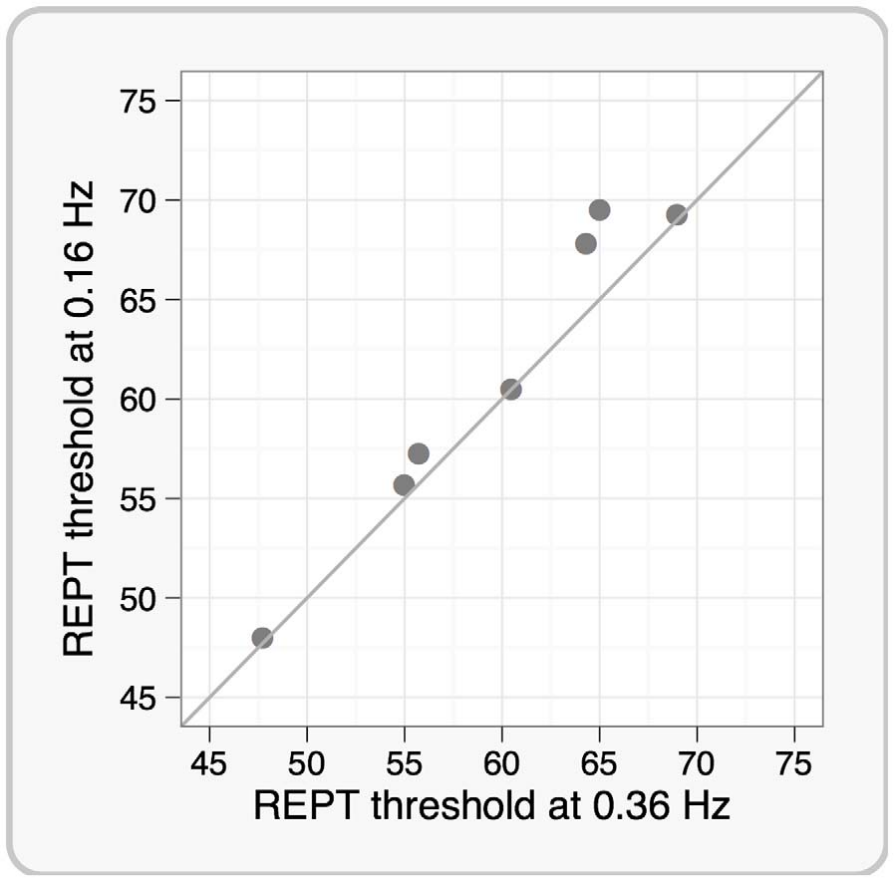
The effect of stimulation frequency on phosphene thresholds using REPT for 7 participants. This scatterplot shows mean phosphene thresholds for each participant estimated using REPT with the average stimulation frequency of 0.36 Hz on the horizontal axis and 0.16 Hz on the vertical axis. The diagonal line indicates where the two estimates are equivalent. No statistically significant difference between the slow and fast staircases was found.

As just described, an advantage of REPT using the Rapid2 Matlab toolbox we have developed is the speed and efficiency with which a staircase can be run. However, it is possible that the low inter-pulse interval may affect the PT because TMS pulses are delivered at a relatively high frequency. That said, the average stimulation frequency in our experiment was 0.36 Hz (30 pulses in 83 sec), which is lower than the single-pulse stimulation frequency used in Desmurget et al. [50] and conforms to the safety guidelines provided in Wassermann et al. [42] and Rossi et al. [43]. Nonetheless, lower stimulation frequencies are commonly used for estimating phosphene thresholds. To test whether the 0.36 Hz stimulation frequency modulates phosphene thresholds estimated with REPT, we compared the PTs obtained using REPT across two different inter-pulse interval settings. In 7 participants, we compared four REPT thresholds using the original average pulse frequency of 0.36 Hz with PTs based on four staircases using a lower pulse frequency of 0.16 Hz. The scatterplot of individual results based on the mean of four REPT staircases for each stimulation condition are shown in Figure 5. For 5 of the 7 participants, the PTs were almost identical for the two procedures (the diagonal line in Figure 5 shows where the two estimates are equivalent), whereas the slower staircase produced a slightly higher estimate of PT in two participants. There was no statistically significant difference between the PTs under the two conditions (*t*(6) = 2.26, *p* = 0.064, the difference between mean thresholds was 1.5).

## Discussion

Overall, the results indicate that REPT provides an improvement over MOBS, which is a commonly used procedure for estimating the PT [31]. We found that REPT is more accurate, reliable and faster than MOBS. REPT also presents a methodological improvement because it uses the Ψ Bayesian staircase procedure [37], which employs a solid mathematical model to estimate psychophysical thresholds.

Initially used to investigate motor physiology, TMS has been embraced by other research domains such as neurophysiology, cognition, emotion, and perception [2], [3], [51]. As applications of TMS continue to develop, many research questions will require more accurate threshold measurements. Indeed, it has been shown that as little as 5% change in stimulator output relative to the PT can result in significant changes in visual perception [17].

The accurate estimation of motor thresholds has become a focus of a few recent publications. The recognition of the bias inherent in using the standard Rossini-Rothwell procedure [21], [28] to estimate MTs lead to an implementation of a staircase procedure to compute those thresholds [52], [53]. The procedure, however, was greeted with scepticism by the TMS researchers working in the clinical field [35], possibly due to a lack of transparency in the actual implementation of the staircase software by Awiszus [52]. A more recent paper proposed an implementation of a Bayesian staircase procedure that estimates the MT in as few as 7 trials [54]. This new procedure has also been criticised as not accurate enough due to a liberal termination criterion [55]. The Ψ staircase used in REPT is set to terminate after 30 trials, as suggested by Kontsevich and Tyler [37]. Here we take a conservative approach aimed at maximising threshold precision. The 30-trial termination rule has been shown to produce threshold estimates with 2 dB precision, in the case of 2-alternative-forced-choice task, which is a common level of precision in psychophysical experiments [37]. Moreover, while the MT can be objectively quantified through MEP amplitudes, the PT depends on a subjective report of a participant which is likely to introduce more variability. Having a conservative termination criterion can therefore benefit the estimation of PTs. However, future studies can explore the possibility of selecting different termination criteria which can provide an accurate PT estimate in fewer trials.

It is a concern that head and coil motion during measurements can influence the estimation of MTs [56] as well as PTs. As the (resting) MT is measured using MEP amplitudes, it does not require verbal responses. On the other hand, PT measurements are commonly based on the participant's verbal report of detected phosphenes which can result in head and coil motion. To counteract this, REPT lets the participant respond using a keyboard which reduces the possibility of head motion. Keyboard responses in REPT coupled with 3D stereotaxic neuronavigation system significantly reduce head and coil motion during the thresholding procedure which can assist in achieving more accurate and reproducible PT measures across different studies.

It is worth noting that PTs might be influenced by testing participants with eyes open versus closed [57], or by different levels of dark adaptation [58], [59]. We always tested participants with their eyes closed, but allowed them to open their eyes between staircase blocks, thereby maintaining a relatively stable level of dark adaptation across the experiment. It is beyond the scope of this paper to consider the impact of differing levels of adaptation or different testing protocols, but we do consider it important that experimenters control these variables within and between participants.

While beyond the scope of the results reported in this paper, REPT presents a possibility to standardise protocols for estimating the PT between different research groups. Most existing procedures for estimating TMS thresholds involve manually adjusting the pulse intensity and the pulse is triggered by the experimenter after warning the participant about the pulse, such as when using the MOBS procedure. REPT, on the other hand, requires minimum intervention from an experimenter during the thresholding procedure because it programmatically controls the Magstim Rapid^2^ stimulator to set the intensity and deliver a pulse. The timing of the pulse delivery is controlled by the participant which leaves the participant better prepared to receive the pulse. This will reduce uncertainty or variability in the participant's response to the pulse compared to trials when the experimenter decides to deliver a pulse and occasionally catching the participant unprepared. Also, reducing the experimenter's direct involvement in the threshold estimation procedure can increase the reproducibility of PT estimates because different experimenters can be a significant source of variability for these types of measurement [39]. Thus giving the control of pulse delivery to the participant, and using a computer to adjust levels of TMS intensities, will likely to improve reproducibility and standardisation of PT measurements. However, this will require more systematic and thorough comparison of different automated thresholding methods. We can expect that an automated MOBS procedure will generally be faster than REPT because fewer pulses are required to converge on a threshold. However, we would also expect that REPT, based on a more robust psychometric measurement model, would provide more accurate and reliable thresholds, as we demonstrated here.

To conclude, the procedure we present here, REPT, represents an improvement over another commonly used procedure for estimating the PT in accuracy, reliability, and speed. Being implemented in Matlab, REPT has an intuitive graphical user interface and its source code is freely available for use and scrutiny of potential users, whose feedback and suggestions can further improve the procedure and help in developing a more standardised approach for measuring the PT to further improve qualities of TMS studies [56].
